# Increasingly capable at the ripe old age? Cognitive abilities from 2004 to 2013 in Germany, Spain, and Sweden

**DOI:** 10.1371/journal.pone.0254038

**Published:** 2021-07-01

**Authors:** Julia Grasshoff, Johannes Beller, Beatrice G. Kuhlmann, Siegfried Geyer

**Affiliations:** 1 Medical Sociology Unit, Hannover Medical School, Hannover, Germany; 2 Department of Psychology, School of Social Sciences, University of Mannheim, Mannheim, Germany; Clinca Geriatrica, ITALY

## Abstract

**Background:**

Life expectancy is increasing in most high-income countries, but gains in life years are maximized if spent in good health and if cognitive abilities are maintained until old age. Age-related decline of cognitive abilities does nevertheless occur, but the pace of decline is decisive. This was the starting point for our study that aims to examine cohort effects of cognitive aging in women and men in Germany, Spain and Sweden by analyzing changes from 2004 to 2013 by estimating cohort effects within age groups starting from the age of 50 years.

**Methods:**

A cohort study was conducted that was based on data of the surveys 2004 (N = 6,081) and 2013 (N = 8,650) from the Survey of Health, Ageing and Retirement in Europe (SHARE). The analyses were based on data of female and male respondents aged 50 years and older. Age-specific means of verbal fluency and delayed recall from the German, Spanish and Swedish samples were the cognitive domains considered in the study.

**Results:**

In both domains of cognitive ability the achievements in the later surveys were higher than in the earlier ones. This was found in all countries, abut achievement levels increased markedly in the German and the Spanish samples, while the scores of the Swedish samples were not significantly different.

While the highest scores were found for Sweden, Germany ranked in the middle and the lowest scores were found in the Spanish samples. Over time, the scores of the German samples approached those of Sweden.

**Conclusions:**

From the first to the second survey, improvements of older adults’ cognitive abilities were found for all countries considered. This may indicate improvements of the underlying educational systems, but also increasingly stimulating general living conditions.

## Background

Preserving cognitive abilities until old age is a main prerequisite for maintaining good health. Studies have shown that the lifetime spent in states of disease and disability was decreasing at the same pace or even faster than life expectancy was increasing [1,2]. In order to uphold health-promoting behavior until older age, cognitive abilities have to be maintained. However, cognitive abilities are declining with increasing age and this process is proceeding with considerable inter-individual variability depending on the cognitive domain considered [3,4]. Until the sixth decade of life, cognitive performances were reported as quite stable, only from the sixth decade on decline is measurable. From the eighth decade of life on, deterioration has been accelerating in all domains except verbal abilities which have been declining at a slower pace [4].

Studies have consistently reported improvements of measured intelligence and cognitive abilities from one generation to the following one. This development became known as the “Flynn-effect” that was described in particular for Germany, France, Israel, Japan, Kenia and the Netherlands, while smaller increases were reported for Brazil, Ireland, New Zealand, Great Britain, and the USA [5–9]. In populations with comparably low levels of cognitive abilities, increases were particularly high, while for Norway and Sweden, stagnation at a very high level was reported. Against the backdrop of studies from Scandinavia, it was discussed whether a ceiling effect exists where further growth is proceeding slowly, or where cognitive abilities are not growing further [10]. This applies to memory, comprehension, spatial orientation, fluid reasoning, calculating capacity, and verbal fluency.

Increments of cognitive abilities were already found in surveys conducted seven years apart [4,11]. For the time between 1990/93 and 2013/14, a German study reported an increase of fluid intelligence in 70 to 98- year old women and men [12], and a second one covering the years 2006 to 2016 confirmed these findings for women and for men aged 50 to 70 years [13]. Both studies used the Wechsler Test [14] for assessing the speed of cognitive processing. A meta-analysis based on pooled data from Germany reported an increase of 3.5 IQ-points per decade for 1971 to 2007 [15]. Also for Sweden, cognitive increments in terms of logical reasoning and spatial orientation were reported for 70-, 75- and 79 year-olds for the birth cohorts 1901, 1906 and 1930 [16]. There are also countries for which no or a low number of studies are available, e.g. for Spain [17].

Direct comparisons between countries have rarely been published. In a meta-analysis, no achievement levels but only increments were reported as they were based on findings from different types of tests [18]. Formanek et al. [19] merged data from Scandinavian, European-Mediterranean, and Western-European countries by using verbal fluency and memory performance as cognitive domains. The highest performance levels were reported for Scandinavia, while Mediterranean-European countries ranked lowest. The annual age-related decrement was highest in Scandinavia and it was lowest in Mediterranean-European countries, i.e., decline was faster if the maximum achievement levels were higher. These findings point towards cognitive reserve that can be considered as abilities that have been acquired over the life course by school education, but also to the complexity of environments individuals are exposed to [20,21]. Regional differences were also examined by Skirbekk et al. [22] and Weber et al. [23] using the same database by comparing subpopulations in terms of verbal fluency, memory and calculating capacity, but again at the level of regions instead of comparing countries. Similar findings were reported by Cadar et al. [17] who compared women and men aged 65+ from European countries with respect to memory performance using survey data from 2004 and 2015. Again, Spanish respondents ranked relatively low, German respondents ranked higher, and Swedish ones ranked highest. However, this study did not focus on between-country comparisons, and country scores were not tested for systematic differences. The available findings comparing Germany and Sweden pointed towards a Flynn-effect [7,8] in terms of fluid and crystallized intelligence, i.e., cognitive abilities based on standardized tests of the same age cohorts were improving over time.

To summarize, the bulk of research refers to selected groups, and comparisons between countries have combined time periods and countries instead of differentiating by age groups, time periods, and countries.

Our study aims to examine cohort effects of cognitive aging in women and men from Germany, Spain and Sweden by analyzing changes from 2004 to 2013 by estimating cohort effects of age groups. Endpoints are verbal fluency as a measure of executive functioning and delayed recall as a measure of episodic memory. These two central domains of cognitive ability are related to fluid intelligence and susceptible to age-related decline [24]. The findings of our study would add to the available evidence on cohort effects in terms of fluid intelligence in the following ways:

- Comparisons between age groups and between countries were beginning at the age of 50 while earlier reports started at the age 65 or 70.
- Cases of possible dementia are excluded after having screened the samples with the Mini-Mental-Status Examination (MMSE) [25].
- Comparisons are performed with smaller age intervals, what makes it possible to analyze continuous developments of cognitive abilities over time.

Against the backdrop of the considerations above, three research questions will be dealt with:

1. Does age-dependent decline of cognitive abilities in terms of delayed recall and verbal fluency occur from the fifth decade of life into old age?
2. Do younger cohorts reach higher levels of cognitive abilities in the two above mentioned domains than older ones, and does this occur likewise in Germany, Sweden, and in Spain?
3. Do Germany, Sweden, and Spain differ with respect to cognitive ability levels, and does age-related decline proceed dependent on the country considered?

## Methods

Our study was based on data of the Survey on Health, Ageing and Retirement in Europe (SHARE) that started in 2004 [26]. SHARE is a multidisciplinary longitudinal study set up to collect microdata on health and illness of individuals aged 50 years and older. The content of the dataset is coordinated with the Health and Retirement Study and the English Longitudinal Study of Ageing. SHARE-data are collected for scientific purposes and available on request (http://www.share-project.org/data-access.html). The authors had no special access privileges, and the data were accessed in the same way as stipulated by the owner.

We used data from Germany, Sweden and Spain from two survey waves conducted in 2004 (first wave) and 2013 (wave five) in order to examine cohort effects by comparing the same age groups.

Before performing the statistical analyses, the number of cases was reduced by several reasons in order to exclude effects that may distort the age and cohort effects we wanted to examine: At the first step, the sample was confined to respondents who participated in the survey for the first time. In earlier studies it was shown that repeated testing may cause training effects thus leading to increased achievement levels [13,27,28]. This applies for short intervals between measurements as well as for longer time periods like seven years as reported in the Seattle Longitudinal Study [4] (pp.29;216). At the second step respondents with missing values on at least one cognitive test were excluded in order to perform all analyses on the basis of the same respondents. We also decided against imputation as the number of missing values was low. At the third step respondents who performed lower than the maximum MMSE-score [29] were also excluded as this may indicate early stages of dementia. Finally, only cases with complete information on cognitive tests and complete demographic data were considered.

### Cognitive measures

Verbal fluency and episodic memory are two domains of fluid intelligence that were assessed as part of computer- or telephone- administered surveys. ***Verbal fluency*** was measured by listing as many animal names as possible within a minute. Thus, the range of values may vary between 0 and a theoretically upper end. Double or non-existing animal names were not counted, and every correct naming was counted as one point. ***Delayed recall*** as a measure of memory was assessed by remembering a list of ten words presented by an interviewer after having performed a distracting task. Again, any correctly remembered word was given one point. Thus, the range of values ranges between 0 and 10. The **Mini-Mental-Status-Examination (MMSE)** was used in order to exclude cases of possible dementia [29]. In SHARE, an abridged version was used that consists of four questions on respondents’ spatial and temporal orientation. Every correct response was given one point, and the data of respondents with a score lower than four were excluded.

### Data analysis

After having inspected the descriptive data over countries and survey waves, multiple regression analyses were performed by using age, gender, wave and age x wave- interactions with each of the two cognitive domains as dependent variables. Confidence intervals were estimated by means of 2000 bootstrap samples in order to obtain normally distributed residuals [30]. Age was divided in intervals of five years: 50–54, 55–59, 60–64, 65–69, 70–74, 75–79, 80–84, 85–89, and 90+. In order to test for pairwise differences between countries, T-Tests were applied by setting an α-level of 5% for each age group. As this made it necessary to perform a large number of tests, the Bonferroni-Correction was applied. It takes the increasing probability of type-1- error into account by adjusting the α-level to the number of tests performed. The corresponding formula is

$$\alpha_{\text{corrected}}{=\alpha }_{\text{chosen a priori}}\text{/n,}$$

where α_corrected_ is the corrected a-level after correction, α_chosen a priori_ is the α-level chosen for accepting a test difference as statistically significant, and n = number of comparisons.

STATA 16 MP was used for all analyses [31].

## Results

### Exclusions

Exclusions directed towards prevention of retest effects led to the reduction of 2914 German respondents from the fifth survey, 3604 cases from the Spanish and 4763 cases from the Swedish sample who had participated in at least one of the preceding waves one, two, or four. The third wave was not considered as no cognitive tests were applied. The results of exclusions due to the occurrence of missing values and due to suspected cases of dementia are depicted in Table 1.

**Table 1:** Exclusions of cases due to missing data (verbal fluency, delayed recall, MMSE*) and due to suspected dementia (MMSE-score <4).

**Numbers of exclusions due to missing data (verbal fluency, delayed recall, MMSE)**		
Wave 1	Germany	75
	Sweden	64
	Spain	79
Wave 5	Germany	99
	Sweden	59
	Spain	297
**Exclusions due to suspected dementia (MMSE <4)**		
Wave 1	Germany	343
	Sweden	316
	Spain	533
Wave 5	Germany	473
	Sweden	284
	Spain	514

* MMSE: Mini-Mental-Status-Examination.

### Basic characteristics of the study samples

The basic characteristics of the three subsamples that entered the final analyses are depicted in Table 2. For all analyses the case numbers were sufficient except for the oldest old respondents.

**Table 2:** Basic distributions of the variables used for analysis by country and survey wave after restriction to cases who participated in SHARE for the first time.

	Germany		Spain		Sweden	
	Survey 2004	Survey 2013	Survey 2004	Survey 2013	Survey 2004	Survey 2013
Sample size	1803	3871	1663	2497	2615	2282
Sex: female	52.60%	53.20%	56.10%	51.50%	54.50%	53.70%
male	47.40%	46.80%	43.90%	48.50%	45.50%	46.40%
Age in years, means (Sd)	64.6 (9.3)	63.5 (9.8)	64.7 (9.7)	65.6 (10.1)	64.6 (9.3)	63.5 (9.8)
Age groups in years, N/%						
50–54	326/18.1%	884/22.8%	296/17.8%	381/15.3%	432/16.5%	265/11.6%
55–59	250/13.9%	717/18.5%	302/18.2%	468/18.7%	550/21.0%	355/15.6%
60–64	351/19.5%	664/17.2%	268/16.1%	428/17.1%	483/18.5%	416/18.2%
65–69	351/19.5%	530/13.7%	260/15.6%	392/15.7%	414/15.8%	505/22.1%
70–74	219/12.2%	466/12.0%	247/14.9%	288/11.5%	299/11.4%	323/14.2%
75–79	190/10.5%	335/8.7%	169/10.2%	244/9.8%	239/9.1%	213/9.3%
80–84	85/4.7%	170/4.4%	77/4.6%	187/7.5%	116/4.4%	137/6.0%
85–89	23/1.3%	85/2.2%	32/1.9%	93/3.7%	67/2.6%	56/2.5%
90–95	8/0.4%	19/0.5%	9/0.5%	16/0.6%	14/0.5%	12/0.5%
95+	0/0%	1/0.03%	3/0.2%	0/0%	1/0.04%	0/0%
Verbal fluency; mean (Sd)	19.9 (6.7)	22.4 (6.9)	15.3 (5.8)	17.4 (7.2)	23.3 (6.9)	23.3 (6.9)
Delayed recall; mean (Sd)	3.71 (1.8)	4.28 (2.2)	2.5 (1.8)	3.2 (1.9)	4.3 (2.0)	4.3 (2.0)

In the following analyses, gender was taken into account, but no separate analyses are presented for women and for men. The reason for this decision was the absence of gender differences in preparatory analyses with our data. With respect to the scaling of the two cognitive tests, gender effects (if they emerged at all) were rather small as documented in the following results of the regression analyses.

### Age-dependent decline of cognitive abilities over countries

In the surveys depicting **Germany**, *verbal fluency* decreased monotonously with age by using the youngest age group (50–54 years) as reference category. The same occurred for *delayed recall*, but the findings from the age above 90 years were based on small case numbers. This applies to survey 2004 as well as to 2013, albeit both were starting from different levels of cognitive ability (Tables 3 and 4). For **Spain**, age-dependent loss of cognitive ability emerged in both survey years and in both domains of cognitive ability (Table 3). For **Sweden**, the same development was found, thus the general finding of cognitive decline with age applies to all countries considered.

**Table 3:** Effects of age, sex and survey wave on verbal fluency and delayed recall for each country compared by OLS- regression; confidence intervals estimated with 2000 bootstrap samples.

	Verbal fluency: Germany				Delayed recall: Germany			
Variable	Coeff.	StE	p	95% CI; lower/upper limit	Coeff.	StE	p	95% CI; lower/ upper limit
**Age**: 50–54	Ref. = 1	--	--	--	Ref. = 1	--	--	--
55–59	-0.50	0.28	0.08	-1.04/ 0.05	-0.35	0.08	<0.001	-0.51/ -0.19
60–64	-1.71	0.28	<0.001	-2.25/ -1.16	-0.84	0.0	<0.00	-1.00/ -0.68
65–69	-2.15	0.30	<0.001	-2.72/ -1.58	-1.02	80.0	1<0.00	-1.19/ -0.86
70–74	-3.36	0.31	<0.001	-3.97/ -2.74	-1.45	90.0	1<0.00	-1.64/ -1.28
75–79	-5.01	0.34	<0.001	-5.68/ -4.34	-1.80	90.1	1<0.00	- 2.00/- 1.61
80–84	-6.59	0.45	<0.001	- 7.47/ -5.71	-2.44	00.1	1<0.00	-2.70/ -2.18
85–89	-8.48	0.65	<0.001	-9.76/ -7.19	-2.92	30.1	1<0.00	-3.30/ -2.50
90+	-9.69	1.27	<0.001	- 12.16/ -7.20	-3.41	90.37	1<0.001	- 4.14/ -2.68
**Sex**:Male	Ref. = 1	--	--	--	Ref. = 1	--	--	--
Female	-0.25	0.17	0.14	-0.60/ 0.08	0.35	0.05	<0.001	0.25/ 0.45
**Survey**:2004	Ref. = 1	--	--	--	Ref. = 1	--	--	--
2013	2.28	0.19	<0.001	1.91/ 2.64	0.50	0.05	<0.001	0.39/ 0.60
**Constant**	22.32	0.25	<0.001	21.82/ 22.82	4.46	0.07	<0.001	4.32/ 4.61
	**Verbal fluency: Spain**				**Delayed recall: Spain**			
**Age**: 50–54	Ref. = 1	--	--	--	Ref. = 1	--	--	--
55–59	-0.57	0.34	0.09	-1.23/0.09	-0.33	0.09	<0.001	-0.50/ -0.15
60–64	-1.11	0.35	0.001	-1.79/ -0.44	-0.50	0.09	<0.001	-0.69/ -0.32
65–69	-2.37	0.35	<0.001	-3.06/ -1.68	-0.76	0.09	<0.001	-0.95/ -0.58
70–74	-3.01	0.40	<0.001	- 3.73/ -2.28	-1.14	0.10	<0.001	-1.34/ -0.95
75–79	-4.82	0.40	<0.001	- 5.60/ -4.04	-1.60	0.10	<0.001	-1.80/ -1.38
80–84	-5.36	0.46	<0.001	- 6.27/ -4.45	-1.90	0.13	<0.001	-2.14/ -1.66
85–89	-6.84	0.62	<0.001	-8.06/ -5.62	-2.36	0.17	<0.001	-2.69/ -2.03
90+	-6.86	1.30	<0.001	-9.41/ -4.32	-1.97	0.35	<0.001	-2.66/ -1.28
**Sex**:Male	Ref. = 1	--	--	--	Ref. = 1	--	--	--
Female	-1.02	0.20	<0.001	-1.41/ -0.63	0.05	0.05	0.32	-0.05/0.16
**Survey**:2004	Ref. = 1	--	--	--	Ref. = 1	--	--	--
2013	2.31	0.20	<0.001	1.92/ 2.72	0.80	0.05	<0.001	0.65/ 0.87
**Constant**	17.83	0.30	<0.001	17.25/ 18.42	3.17	0.08	<0.001	3.02/ 3.33
	**Verbal fluency: Sweden**				**Delayed recall: Sweden**			
**Age**: 50–54	Ref. = 1	--	--	--	Ref. = 1	--	--	--
55–59	-0.30	0.33	0.38	-0.94/ 0.36	0.33	0.09	<0.001	-0.15/ -0.16
60–64	-0.82	0.33	0.01	-1.47/ -0.17	0.52	0.09	<0.001	-0.70/ -0.34
65–69	-2.19	0.33	<0.001	-2.84/ -1.54	0.80	0.09	<0.001	-0.98/ -0.62
70–74	-3.36	0.36	<0.001	-4.07/ -2.64	1.32	0.10	<0.001	-1.52/ -1.13
75–79	-5.49	0.40	<0.001	-6.27/ -4.71	1.60	0.11	<0.001	-1.81/ -1.39
80–84	-7.00	0.48	<0.001	-7.94/ -6.04	2.20	0.13	<0.001	-2.46/ -1.95
85–89	-8.53	0.64	<0.001	-9.79/ -7.26	2.87	0.17	<0.001	-3.23/ -2.53
90+	-10.08	1.31	<0.001	-12.66/ -7.50	2.96	0.36	<0.001	-3.66/ -2.26
**Sex**:Male	Ref. = 1	--	--	--	Ref. = 1	--	--	--
Female	0.10	0.19	0.59	-0.27/ 0.47	0.63	0.05	<0.001	0.53/ 0.73
**Survey**:2004	Ref. = 1	--	--	--	Ref. = 1	--	--	--
2013	0.13	0.19	0.49	-0.24/ 0.50	0.28	0.05	<0.001	0.18/ 0.38
**Constant**	24.43	0.28	<0.001	24.87/ 25.98	4.53	0.08	<0.001	4.38/ 4.68

**Table 4:** Analyses of differences between surveys for Germany, Spain and for Sweden by cognitive domain based on T-tests after Bonferroni-correction.

Germany												
	Verbal fluency						Delayed recall					
	Survey wave 2004		Survey wave 2013				Survey wave 2004		Survey wave 2013			
Age	*M*	*Sd*	*M*	*Sd*	t	p	*M*	*Sd*	*M*	*Sd*	t	p
50–54	22.47	6.88	24.35	6.80	4.24	< .05*	4.49	1.84	5.23	2.05	5.71	< .05*
55–59	21.64	7.05	23.98	6.53	4.80	< .05*	4.08	1.84	4.88	1.94	5.69	< .05*
60–64	20.50	6.28	22.74	7.06	4.99	< .05*	3.67	1.72	4.29	1.93	3.38	< .05*
65–69	19.38	6.38	21.87	6.65	7.35	< .05*	3.48	1.69	3.90	2.12	4.12	< .05*
70–74	17.93	5.84	21.53	6.37	7.09	< .05*	3.20	1.81	3.68	2.01	3.01	< .05*
75–79	17.36	5.47	19.36	6.06	3.76	< .05*	3.06	1.75	3.22	1.93	.93	.18
80–84	14.92	5.07	18.20	6.26	4.19	< .05*	2.51	1.49	2.58	1.95	.29	.39
85–89	14.96	5.42	15.64	5.15	.55	.29	2.48	1.41	2.05	1.83	- 1.05	.85
90–99	14.38	4.72	13.95	4.60	-.22	.59	1.36	1.51	1.74	1.70	.52	.30
Spain												
50–54	16.87	5.56	19.82	5.56	-6.10	< .05*	3.26	1.94	3.92	1.82	-4.56	< .05*
55–59	16.47	5.57	19.20	7.89	-5.23	< .05*	3.00	1.78	3.55	1.82	-4.12	< .05*
60–64	15.88	5.64	18.71	8.02	-5.05	< .05*	2.57	1.57	3.54	1.84	-6.97	< .05*
65–69	15.15	7.22	17.11	6.31	-3.67	< .05*	2.32	1.57	3.28	1.74	-7.13	< .05*
70–74	14.22	4.72	16.71	6.01	-5.28	< .05*	1.96	1.51	2.90	1.73	-6.69	< .05*
75–79	12.71	4.11	14.66	6.03	-3.65	< .05*	1.60	1.41	2.38	1.62	-5.03	< .05*
80–84	13.13	4.44	13.71	5.50	-0.82	.21	2.66	1.79	2.91	1.88	-1.07	.14
85–89	11.56	5.14	12.37	6.34	-0.65	.26	1.91	1.84	2.30	1.89	-1.16	.12
90–99	12.78	4.89	11.44	4.19	0.73	.76	1.57	1.65	2.58	2.35	-1.28	.11
Sweden												
50–54	25.56	6.89	25.50	7.38	0.10	0.54	4.86	1.66	5.25	1.84	-2.88	< .05*
55–59	24.95	7.09	25.70	6.15	-1.64	0.05	4.51	1.75	4.93	1.92	-3.40	< .05*
60–64	24.71	6.76	24.74	6.77	-0.07	0.47	4.41	1.69	4.54	1.86	-1.05	< .05*
65–69	23.39	6.45	23.35	6.46	0.11	0.54	4.18	1.69	4.27	1.89	-0.79	.21
70–74	21.90	6.33	22.46	6.24	-1.11	0.13	3.56	1.85	3.79	1.95	-1.51	.07
75–79	20.21	6.46	19.87	6.12	6.12	0.72	3.12	1.88	3.68	1.88	-3.19	< .05*
80–84	19.29	6.65	17.93	5.48	1.79	0.96	2.66	1.79	2.91	1.88	-1.17	.14
85–89	16.57	6.65	17.54	5.61	-0.86	0.20	1.91	0.25	2.30	1.89	-1.07	.12
90–99	14.21	5.92	16.92	4.23	-1.31	0.10	1.57	1.65	2.58	2.35	-1.28	.11

### Within-country differences of achievement levels between survey waves by age groups and countries

Looking at changes over time by country, an overall difference of Diff_2004/2013_ = 2.28 points emerged for *verbal fluency* between the two German surveys. In the Spanish surveys, the difference (Diff_2004/2013_ = 2.31) was about the same size. Thus in these two countries, achievement levels had improved over time. For Sweden, no difference between samples emerged as the overall difference was only Diff_2004/2013_ = 0.13 points which is rather small given the open-ended scale (see Table 4 for the numeric scores). Looking at changes by age groups, the differences between the German samples were statistically significant for the age groups until 84 years, and the same held in the Spanish samples up to the age of 79, but at lower levels if compared with Sweden and Germany. Sweden was different as verbal fluency did not improve over time, but this has to be considered against the backdrop of high achievement levels in all age groups.

For *delayed recall*, statistically significant increments from the earlier to the later surveys emerged. Depending on the scaling of 0 to 10 points, smaller changes compared to verbal fluency occurred in absolute terms. This held for the samples of all countries with the highest changes having occurred in the Spanish samples (Table 4). In the German surveys, increments emerged up to the age of 74 years, while in the Spanish ones, statistically significant changes occurred up to the age of 79 years. In the Swedish samples, changes also tended upwards (Table 2), but smaller in magnitude than in the other two countries. Changes were statistically significant up to the age of 64 years and again for the age group 75 to 79 years (Table 4).

### Differences between achievement levels between countries by age groups and survey

**Fig 1:**
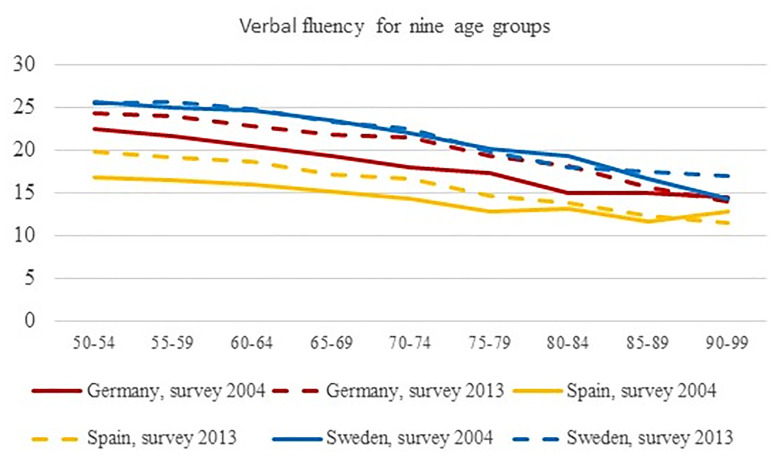
Scores of verbal fluency (range from 0 without upper end) for Germany, Spain and Sweden for surveys 2004 and 2013 for nine age groups. The lines between the point estimates do not indicate linear changes, they are shown to indicate differences between measurements.

**Fig 2:**
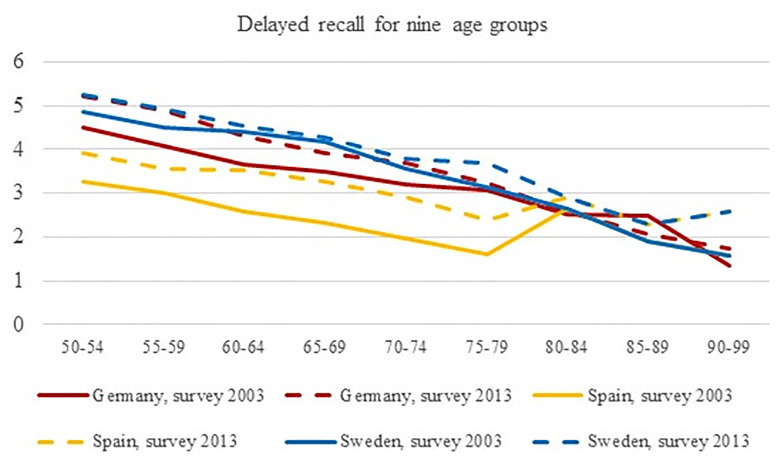
Scores of delayed recall (memory; range from 0 to 10) for Germany, Spain and Sweden for surveys 2004 and 2013 for nine age groups. The lines between the point estimates do not indicate linear changes, they are shown to indicate differences between measurements.

Figs 1 and 2 graphically depict the developments of cognitive abilities over time by cognitive domain. Detailed results of pairwise tests between countries by age groups are displayed in Table 5. The Swedish samples had the highest achievement levels in both surveys, and age-dependent decline was more pronounced than in the samples of the two other countries. Achievement levels of the German samples were comparably lower than those of Sweden, while the Spanish samples reached consistently lower scores than the other ones with a less pronounced decline. For delayed recall, the achievement levels of the three countries were converging for the age groups beyond the age of 79, thus pointing towards relatively strong declines in the Swedish samples and flatter curves for the Spanish samples.

**Table 5:** Results of pairwise tests of differences between countries (Germany, Spain, and Sweden) for verbal fluency and delayed recall by age groups.

**Verbal fluency**																		
**2013**																		
	Germany vs. Spain						Germany vs. Sweden						Spain vs. Sweden					
	Germany		Spain				Germany		Sweden				Spain		Sweden			
Age	***M***	***Sd***	***M***	***Sd***	t	p	***M***	***Sd***	***M***	***Sd***	t	p	***M***	***Sd***	***M***	***Sd***	t	p
50–54	24.35	6.80	19.82	5.56	10.88	<.05*	24.35	6.80	25.50	7.38	-2.34	.019*	19.82	5.56	25.50	7.38	10.12	<.05*
55–59	23.98	6.53	19.20	7.89	11.24	<.05*	23.98	6.53	25.70	6.15	-4.14	<.05*	19.20	7.89	25.70	6.15	12.85	<.05*
60–64	22.74	7.06	18.71	8.02	8.74	<.05*	22.74	7.06	24.74	6.77	-4.59	<.05*	18.71	8.02	24.74	6.77	11.78	<.05*
65–69	21.87	6.65	17.11	6.31	11.66	<.05*	21.87	6.65	23.35	6.46	-2.92	.004*	17.11	6.31	23.35	6.46	14.51	<.05*
70–74	21.53	6.37	16.71	6.01	10.32	<.05*	21.53	6.37	22.46	6.24	-2.03	.038*	16.71	6.01	22.46	6.24	11.58	<.05*
75–79	19.36	6.06	14.66	6.03	9.24	<.05*	19.36	6.06	19.87	6.12	-0.95	.355	14.66	6.03	19.87	6.12	9.15	<.05*
80–84	18.20	6.26	13.71	5.50	7.21	<.05*	18.20	6.26	17.93	5.48	0.40	.684	13.71	5.50	17.93	5.48	6.83	<.05*
85–89	15.64	5.15	12.37	6.34	3.75	.001*	15.64	5.15	17.54	5.61	-2.07	.034*	12.37	6.34	17.54	5.61	5.03	<.05*
90–99	13.95	4.60	11.44	4.19	1.67	.125	13.95	4.60	16.92	4.23	-1.80	.063	11.44	4.19	16.92	4.23	3.41	<.01*
**Delayed recall**																		
**2004**																		
	Germany vs. Spain						Germany vs. Sweden						Spain vs. Sweden					
	Germany		Spain				Germany		Sweden				Spain		Sweden			
Age	***M***	***Sd***	***M***	***Sd***	t	p	***M***	***Sd***	***M***	***Sd***	t	p	***M***	***Sd***	***M***	***Sd***	t	p
50–54	4.49	1.84	3.26	1.94	8.21	<.05*	4.49	1.84	4.86	1.66	-2.86	.889	3.26	1.94	4.86	1.66	11.97	<.05*
55–59	4.08	1.84	3.00	1.78	6.97	<.05*	4.08	1.84	4.51	1.75	-3.17	.687	3.00	1.78	4.51	1.75	11.96	<.05*
60–64	3.67	1.72	2.57	1.57	9.63	<.05*	3.67	1.72	4.41	1.69	-4.55	.027*	2.57	1.57	4.41	1.69	14.65	<.05*
65–69	3.48	1.69	2.32	1.57	9.89	<.05*	3.48	1.69	4.18	1.69	-4.32	.189	2.32	1.57	4.18	1.69	14.30	<.05*
70–74	3.20	1.81	1.96	1.51	8.08	<.05*	3.20	1.81	3.56	1.85	-2.20	.450	1.96	1.51	3.56	1.85	10.93	<.05*
75–79	3.06	1.75	1.60	1.41	8.67	<.05*	3.06	1.75	3.12	1.88	-0.34	.012*	1.60	1.41	3.12	1.88	8.93	<.05*
80–84	2.51	1.49	2.66	1.79	4.04	<.05*	2.51	1.49	2.66	1.79	-0.66	.119	2.66	1.79	2.66	1.79	4.44	<.05*
85–89	2.48	1.41	1.91	1.84	3.09	.010*	2.48	1.41	1.91	0.25	1.35	.412	1.91	1.84	1.91	0.25	1.77	.05
90–99	1.36	1.51	1.57	1.65	-0.24	.823	1.36	1.51	1.57	1.65	-0.28	.267	1.57	1.65	1.57	1.65	0.02	.98
**Delayed recall**																		
**2013**																		
	Germany vs. Spain						Germany vs. Sweden						Spain vs. Sweden					
	Germany		Spain				Germany		Sweden				Spain		Sweden			
Age	***M***	***Sd***	***M***	***Sd***	t	p	***M***	***Sd***	***M***	***Sd***	t	p	***M***	***Sd***	***M***	***Sd***	t	p
50–54	5.23	2.05	3.92	1.82	10.84	<.05*	5.23	2.05	5.25	1.84	-0.13	.005*	3.92	1.82	5.25	1.84	9.08	<.05*
55–59	4.88	1.94	3.55	1.82	11.80	<.05*	4.88	1.94	4.93	1.92	-0.41	.002*	3.55	1.82	4.93	1.92	10.51	<.05*
60–64	4.29	1.93	3.54	1.84	6.37	<.05*	4.29	1.93	4.54	1.86	-2.13	<.05*	3.54	1.84	4.54	1.86	7.83	<.05*
65–69	3.90	2.12	3.28	1.74	6.26	<.05*	3.90	2.12	4.27	1.89	-1.35	<.05	3.28	1.74	4.27	1.89	8.05	<.05*
70–74	3.68	2.01	2.90	1.73	5.44	<.05*	3.68	2.01	3.79	1.95	-0.76	.028*	2.90	1.73	3.79	1.95	5.92	<.05*
75–79	3.22	1.93	2.38	1.62	5.64	<.05*	3.22	1.93	3.68	1.88	-2.57	.739	2.38	1.62	3.68	1.88	7.84	<.05*
80–84	2.58	1.95	2.91	1.88	3.41	.001*	2.58	1.95	2.91	1.88	-1.53	.497	2.91	1.88	2.91	1.88	4.99	<.05*
85–89	2.05	1.83	2.30	1.89	2.45	.014*	2.05	1.83	2.30	1.89	-0.80	.155	2.30	1.89	2.30	1.89	3.17	<.01*
90–99	1.74	1.70	2.58	2.35	-0.14	.895	1.74	1.70	2.58	2.35	-1.17	.784	2.58	2.35	2.58	2.35	1.04	.32

When changes in terms of *verbal fluency* were reconsidered in terms of T-tests of the same age groups between countries and surveys, consistent differences emerged for comparisons between Germany and Sweden with data from survey 2004. This was not reproduced for survey 2013, thus reflecting upward changes in the German samples and relatively small increments in the Swedish ones. Changes were also statistically significant in comparisons between Spain and Germany, and it has to be kept in mind that the mean scores for Germany and for Spain had increased over the two surveys, thus the initial differences were reproduced at higher mean levels.

For *delayed memory*, age-related differences between Germany and Sweden failed in most cases to become statistically significant in the first survey and in the older groups of survey 2013. For comparisons with Spain, all tests with Germany and Sweden were statistically significant except for the oldest old. This reflects the consistently lower levels of cognitive ability of the Spanish samples.

## Discussion

The first research question addressed whether cognitive decline might become effective already from the age of 50 years and continue until old age. We found that for Germany, Spain and Sweden, cognitive abilities were declining already from the age of 50 years on, and the speed of decline was relatively stable over life years. This corresponds to findings from the Seattle Longitudinal study where cognitive decline was reported before the age of 60 [4]. In our study, decline from the age of 50 on occurred at a similar pace compared to respondents between the age of 65 and 80 which may in part be due to the small age intervals considered, and this had not been reported in earlier studies [3]. Apart from this decline, we also found marked inter-individual variations in respondents of 80 to 85 years of age. Again, the small intervals used in our study made it possible to study gradual changes, and the variation of cognitive abilities indicates that changes may only partly be explained by mere processes of ageing. It is necessary to refer to environmental influences [10], and in the literature, several potential driving forces were identified [32]. During the economically active phase of life, the structure of occupational work is particularly important as complex and demanding activities have been found to predict cognitive ability [33,34]. After retirement, the maintenance of cognitive abilities is dependent on stimulating activities, in particular those associated with active social relationships [35,36].

The second research question addressed whether the achievement levels of the two cognitive domains considered were higher in the more recently assessed cohorts compared to the earlier cohort. Our findings indicate that from 2004 to 2013 the achievement levels had increased in all age groups. This was found for Germany and for Spain, while for Sweden a cohort effect was found only for delayed recall, and the amount of change was smaller than for the other two countries. These findings are in favor of the Flynn-effect, described as improvements of cognitive functions over time [4,5,22]. However, the case of Sweden also demonstrates that after having reached a certain level a ceiling effect may have occurred, i.e., further improvements are small or may not take place at all. It remains an open question whether achievement levels at population level have reached an upper limit or whether further changes of the educational system or the social structure of a country may shift levels upwards. Our results also add to findings from earlier studies by showing that improvements have not only taken place in the older groups of the study population, but over the whole age range covered by our data.

The third research question referred to differences of cognitive levels between countries, and whether decay proceeded differently with increasing age. Over all survey waves and age groups, the achievement levels of Swedish samples had been higher than those of the German and the Spanish ones, although the German samples were catching up. If the three countries are considered as representatives of Scandinavian, Western European, and Mediterranean countries as it had been done in earlier work [22], our findings would be in accordance with studies where Scandinavian samples had been reported to do better than others [22,23]. Our study adds to these findings by reproducing the above-mentioned ranking for memory. The absence of improvements in the Swedish sample may be explained by ceiling effects that may prevent further improvements of the following birth cohorts. It should be noted that this only applies to verbal fluency, but not to delayed recall, thus leaving potentials for improvements. As the scores of the German sample were approaching the Swedish one in 2013, it should be examined whether ceiling effects will occur in future survey waves, and how these developments can be explained. Potential explanations are differences of educational systems. Earlier studies have demonstrated that the level of school education has long-lasting effects until old age [9,37–39]. After having acknowledged the pivotal role of school systems, this should lead to efforts to ensure that all segments of the population are profiting while access to a high level of education should not be taken for granted [40,41].

Against the backdrop of higher intercepts (i.e. higher levels of departure) of cognitive achievements for Sweden and Germany, steeper negative slopes in terms of age and cognitive ability were found for both domains in our data. Spain started at lower achievement levels, but the curve of age-related decline was rather flat. This finding is in accordance with findings reported by Formanek et al. [19] who merged countries into the above-mentioned larger categories Western European, Scandinavian, and Mediterranean. These findings also apply to the three single countries, and the rapid age-related decline from high levels of cognitive ability are in accordance with the concept of cognitive reserve as developed by Stern [42]. Cognitive reserve has to be developed by education and stimulating experiences. Our findings suggest that the mean level of education may differ over countries, although they are generally improving over time. Effects of education and respective enhancements of cognitive ability require all groups of society to gain from improvements of education, but there is also evidence that individuals with higher educational degrees may profit more than those with lower degrees [40]. We did not explore effects of educational qualification as the focus was on international comparisons. Detailed analyses by educational levels would also make it necessary to compare systems of school education between countries, and this is beyond the scope of this paper. For evidence on beneficial effects of school education readers may refer to studies on this topic [39,43–45]. Apart from educational qualification, the development and maintenance of cognitive abilities were reported to be fostered by social [46] and intellectual activities [47], as well as by exercise [48,49].

It had already been mentioned that we did not perform separate analyses for women and for men as differences either did not emerge, or were very small in magnitude. These findings are in line with earlier papers with German data using other databases and other tests [13,50]. Studies comparing international findings did not differentiate between women and men [5], thus our study confirms the findings on the lack of gender differences for Germany, and it adds to the international literature on this topic with respect to Spain and Sweden.

Finally, some limitations of our study have to be acknowledged. In order to examine cognitive aging in healthy populations, individuals with cognitive impairments and dementia were excluded. However, other potential reasons for exclusion such as traumatic brain injury, stroke or medications had not been included in the dataset used for our study. In addition, by excluding repeatedly participating respondents, training effects should be controlled for [4,27]. This approach is recommended, in particular if several survey waves are considered. An associated potential disadvantage is the exclusion of specific groups which might have led to selection effects. It was reported that individuals at higher achievement levels were more likely to participate in follow-ups, while ranking in average or lower levels was associated with lower probability of participation [10]. Thus, achievement levels of cohorts may be overestimated.

Our study was confined to verbal fluency and episodic memory as domains of fluid intelligence. It might have been worthwhile to extend the analyses to other domains, but we finally decided against it because considering more cognitive domains would have inflated the results section further as the presentation of results had to include illustrated comparisons between countries and their educational systems. The second reason refers to the completeness of data. In the Seattle longitudinal study, it was reported that numeric abilities had deteriorated over time [51]. It was planned to consider this dimension, but the large number of respondents with missing data was too high for permitting analyses.

Taken together, we have shown that levels of cognitive ability in adults aged 50 and older had increased over time in terms of delayed recall and verbal fluency, that cognitive decline had already started at the age of 50, and that there were considerable differences in cognitive abilities between the three countries considered. Finally, the amount of decline depends on the levels achieved before which may be taken as a strong argument for striving for high population levels of formal education. So far, one might be satisfied with the findings and expect this development to continue until having reached a plateau where further increments are less likely. There are however findings that indicate that the development may also go into the opposite direction depending on the age cohorts and on the cognitive domains considered [4,41].
